# A qualitative inquiry of food insecurity in Belize

**DOI:** 10.1017/S1368980021002615

**Published:** 2022-04

**Authors:** Laurel D Stevenson, Melissa M Reznar, Elizabeth Onye, Lynna Bendali Amor, Andre J Lopez, Rita DeFour

**Affiliations:** 1 Interdisciplinary Health Sciences, Oakland University, 433 Meadowbrook Road, Rochester, MI 48309, USA; 2 Master of Public Health Program, Oakland University, Rochester, MI, USA; 3 Ministry of Human Development, Social Transformation and Poverty Alleviation, Belmopan, Belize; 4 The Cornerstone Foundation of Belize, San Ignacio, Cayo District, Belize

**Keywords:** Food security, Food access, Qualitative, Belize

## Abstract

**Objective:**

To explore and provide contextual meaning around issues surrounding food insecurity, namely factors influencing food access, as one domain of food security.

**Design:**

A community-based, qualitative inquiry using semi-structured face-to-face interviews was conducted as part of a larger sequential mixed-methods study.

**Setting:**

Cayo District, Belize, May 2019–August 2019.

**Participants:**

Thirty English-speaking individuals (eight males, twenty-two females) between the ages of 18–70, with varying family composition residing within the Cayo District.

**Results:**

Participants describe a complex interconnectedness between family- and individual-level barriers to food access. Specifically, family composition, income, education and employment influence individuals’ ability to afford and access food for themselves or their families. Participants also cite challenges with transportation and distance to food sources and educational opportunities as barriers to accessing food.

**Conclusion:**

These findings provide insight around food security and food access barriers in a middle-income country and provide avenues for further study and potential interventions. Increased and sustained investment in primary and secondary education, including programmes to support enrollment, should be a priority to decreasing food insecurity. Attention to building public infrastructure may also ease burdens around accessing foods.

Food insecurity is uncertain availability or access to high quality, nutritious food, as conceptualised by the FAO’s Food Insecurity Experience Scale^(1,2)^. Globally, one hallmark indicator of food insecurity is undernourishment^(3)^. Undernourishment, as one form of malnutrition in children, can lead to underweight, wasting and growth stunting^(3)^. Longitudinally, these can lead to increased risk of metabolic and CVD in adulthood, decreases in cognitive ability and reproductive performance, and intergenerational health and economic consequences^(4,5)^. Common mental disorders, such as anxiety and depression, have also been linked to food insecurity^(6,7)^. Due to the devastating nature of these consequences combined, the FAO and affiliated organisations have established ending hunger, achieving food security and improved nutrition, and promoting sustainable agriculture by 2030 as one of the Sustainable Development Goals, Goal #2 End Hunger^(8)^. One target of this goal is to ‘ensure access by all people, in particular the poor and people in vulnerable situations, including infants, to safe, nutritious and sufficient food all year round’^(8)^.

Food insecurity can result in both undernourishment due to lack of access to food and overnourishment because of reliance on cheap, calorically dense foods. In Belize, the prevalence of child undernourishment remains higher than other Central American countries^(3)^, and the prevalence of child stunting has held steady at 15·0 % between 2015 and 2018^(3,9)^. Stunting has decreased but still remains above other Central American (12·9 %) and Caribbean nations (8·3 %)^(3)^. At the same time, the prevalence of overweight in Belizean children (7·3 %) is higher than other Central American (6·9 %) and Caribbean nations (7·0 %)^(3,10)^. In the adult population, diabetes (17 %)^(11)^ and obesity (22·4 %) are prevalent^(3)^. As such, Belize experiences a double burden of malnutrition^(10,12)^, but may also experience the so-called ‘Obesity Paradox’, which suggests that food insecurity can increase risk of obesity due to reliance on low-quality, highly processed foods that are often cheaper than healthier options^(13,14)^.

Less developed countries tend to have higher levels of food insecurity^(3)^. Though Belize classifies as a middle-income county, over 40 % of the population lives in poverty, and 15 % lives in extreme poverty^(15,16)^. Belize experienced two major economic slowdowns between 2006 and 2011, coinciding with a rise in the prevalence of undernourishment in the population^(3)^. A 2016 FAO report estimated Belize’s ‘moderate or severe food insecurity’ prevalence (eating less than should due to lack of money or other resources) at 28 %, and approximately 9 % of this group experiences ‘severe food insecurity’ (going an entire day without eating due to lack of money or other resources)^(17,18)^. One recent study indicates that food insecurity in Belize is significantly related to education level^(19)^. This is consistent with documentation that suggests that education is a major factor worldwide^(20)^. However, research on food insecurity in Belize is very limited, and there have been no studies to our knowledge that have involved a thorough exploration of factors related to food insecurity in Belize. For this present study, we conducted a qualitative inquiry in the Cayo District, Belize, to provide an in-depth, contextually rich description of issues leading to and arising from food insecurity. These findings will inform a discussion at the broader environmental and policy levels in Belize and to aid in development of tailored intervention strategies that will ease subsequent health issues.

## Methods

### Design

This qualitative inquiry, conducted in 2019, is the second phase of a larger community-driven sequential mixed-methods study and builds upon the findings from a cross-sectional community-based survey distributed in the Cayo District, Belize in 2017^(19)^. As a follow-up, this present study sought to provide contextual meaning around food security issues in Belize. Thirty in-depth interviews were conducted with Belizean residents of the Cayo District.

### Setting

The Cornerstone Foundation, a Belizean non-governmental, community-based organisation, initiated this study in 2017 to understand more comprehensively needs around food among the community and individuals they serve. The Cornerstone operates as a humanitarian and social service organisation, primarily in the twin towns of San Ignacio and Santa Elena, though its service area extends to the entirety of the Cayo District. With its mandate to protect and care for vulnerable children, one of the Cornerstone’s primary services involves providing hot lunches for local school children; however, this organisation also operates a food pantry from which it feeds hungry families, seniors and individuals facing homelessness or substance abuse issues. With the bulk of its programming focused on supplementing food to those facing food insecurity in its community, Cornerstone staff partnered with an Oakland University faculty member, who has an ongoing working relationship with the organisation, and Oakland University Master of Public Health students to investigate food security issues in its service area.

### Interview protocol and instrument

Participants were recruited using word-of-mouth snowball sampling with the use of a financial incentive equivalent to $20 USD. Word-of-mouth recruitment started with Cornerstone staff as the key informants and dispersed into their social networks and into the community of people they serve. All interviews were conducted in English, lasted approximately 15–30 min, and were recorded by a student who was working with Cornerstone during a practicum placement. Potential participants were provided the contact information of this student at Cornerstone, and willing participants, aged 18 and older, who were food gatekeepers (responsible for food purchasing and food preparation) made interview appointments at the Cornerstone office or other public locations, such as businesses or the local market. Data collection took place until participants repeated similar ideas, and no new themes were emerging from interviews, thus achieving data saturation.

As this research was part of a larger sequential mixed-methods study, formative work was conducted to develop the interview guide and overall protocol. Through cognitive interviewing and in a participatory fashion, Cornerstone staff and several community members reviewed and revised the interview guide to ensure cultural appropriateness, relevancy of topics and high face validity of questions. The interview guide structured three of its domains – availability, access, and utilisation – to mirror three components of food security^(1)^ and was designed using select models of health behaviour theories to elicit responses rooted in these theories^(21)^, namely the Health Belief Model^(22)^, the Social Cognitive Theory^(23)^ and the Theory of Planned Behaviour^(24)^. In building upon previous work^(19)^, these three theories support a line of questioning around perceived barriers, self-efficacy and perceived behavioural control to provide additional insight into factors affecting food security and food access. The primary prompt for the food availability section read, ‘Tell me about your food’. Each question had optional follow-up questions depending on the loquacity of the participant (e.g. Where do you usually buy your food? Do you grow your own food?). The food access domain contained the questions, ‘What things make it easy or hard for people in your community to get access to healthy food?’ and ‘What sort of foods do you and your family prefer? Are you able to eat those preferred foods? Why or why not?’ This domain contained questions that were geared to reveal any potential perceived barriers and facilitators to food access, reflecting a component of the Health Belief Model and the Theory of Planned Behaviour. The food utilisation section asked participants to describe their typical individual or family meals, foods they choose to purchase and methods of food preparation in addition to inquiring whether participants felt in control of eating healthy. This question of control was intended to reveal participant self-efficacy and perceived behavioural control for healthy eating and is rooted in the Social Cognitive Theory and the Theory of Planned Behaviour.

### Analyses

**Fig. 1:**
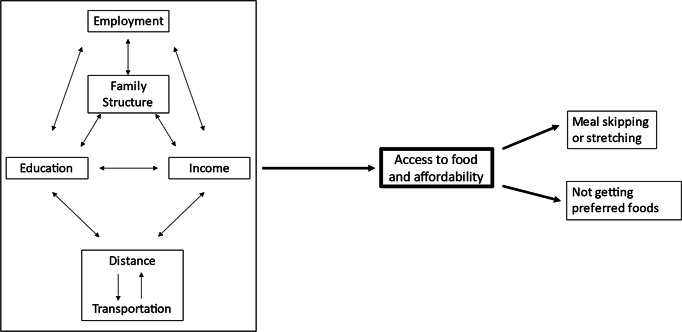
Conceptual map of interpersonal barriers related to food access and food security

Interview recordings were transcribed manually using Microsoft Office and Windows Media Player, with field notes added to the transcripts. Analysis broadly followed the steps outlined by Braun and Clark^(25)^. An interdisciplinary four-member team with backgrounds in public health, health behaviour, nutrition, social work and nursing conducted a thematic analysis through a process of manual coding. Each member read all transcripts and generated initial codes across a series of three identical transcripts, using a blended approach of inductive and theoretical coding. For example, some codes and themes were generated without an existing coding framework, similar to grounded theory where themes are generated from the data themselves^(26)^, while other codes and themes were linked to theoretical frameworks (e.g. distance to food source as a perceived barrier from the Health Belief Model). Codes and potential themes were discussed among members, and two members then (re)coded and collated codes into themes across all interviews. Members continually reviewed and revised codes and themes as a group until agreement was reached. During the overall coding process, Cornerstone staff occasionally provided more context around a code or theme, for example, describing distinct educational costs associated with levels of attendance at different schools in the Cayo District. A conceptual model of themes was generated to describe food access and security in the Cayo District (see Fig. 1). This visual representation helps to provide additional context on how narrative text and themes relate across all interviews.

## Results

Interview participants included thirty individuals who ranged in age from 18 to 70. All were residents of the Cayo District – of San Ignacio, Santa Elena, or a nearby village with public transportation or close enough for walking and bicycling to the town centres. In total, eight men and twenty-two women participated. Participants’ ethnicities included Kriol, Mestizo, Chinese, Garifuna and mixes of ethnicities. Participants were an assortment of single individuals and people with families of varying composition.

These in-depth interviews provide insight into family- and individual-level barriers to food access particularly around the interplay and compounding effects between (i) family composition and individual-level barriers, (ii) income, (iii) education, (iv) employment and (v) issues involving transportation and distance to foods (see Fig. 1). Overall, individuals described an interconnectedness between family composition (overall size, number of children/grandchildren and single- or double-parent households), generating income to support the family, education (sending children to school or being able to attend school themselves) and employment opportunities. Participants also broadly described challenges with transportation and distance to food sources and educational opportunities. Representative quotes are shown in Table 1.

**Table 1:** Themes associated with perceived barriers to food access for Belizeans (*n* 30) living in the Cayo District, Belize, May–August, 2019

Theme	Illustrative quotes
Family structure	
Large family	‘And it depends you know like for example, if you’re a big family it could be hard. You have to eat what you get, you know. If it’s a family of six or seven, you can’t be keeping up that diet as how you want all the nutritious.’ [28, 2019.06.05, 18:54]
	‘Not saying it’s all, but the families that are big numbers, it’s harder for them.’ [P25, female]
Single parenting	‘I would see some kids that around their parents are single parents, like the mother have two or four kids. It’s hard for them to provide a plate of food for each one of them.’ [P8, male]
	‘You know, some of these teenage girls that are mothers, like they don’t have any assistance from the father of the child, you know, because of so many issues going on, like um there’s a lot of young girls that drop out of school.’ [P14, female]
Income, financial status and affordability	
Poverty/low income	‘Sometimes, maybe if I want something else to eat, I don’t…can’t afford to buy that kind of food.’ [P15, female]
	‘…Poverty. Maybe some people can’t afford to buy groceries…’ [P12, female]
	‘Finances is the number one thing. The amount of money people make to get to, to actually pay for food and healthy food um it’s sometimes out of reach for a lot of people. That’s one of the major things.’ [P1, male]
	‘I think it’s too high, because I remember at one time that we could buy affordable stuff but now it’s like you couldn’t barely buy stuff with the money. I’ve seen it happen around a lot.’ [P10, female]
Taxes	‘Well, our government keeps borrowing money and, God knows where the money goes, and they keep taking the taxes up, so prices of the stuff go up, because then the more taxes the stores have to pay, the higher the price of the product have to be. So, I feel like that’s a major factor.’ [P14, female]
	‘A lot of things that is going on here, you know. Teachers asking for raise of pay. And people asking for this and that. And um, the government has to raise tax, so prices has to go up. And then, things that comes in imported might be a little bit expensive too…Sometimes, I get frightened when I go to buy. Because, for example, if you buy canned food you see there’s like 15 to 20 dollars in tax that you pay. Like you’re scared, you know. Even if you go to the stores, you see a lot of sales tax they charge you. So, it’s something pricey. You know, I don’t know why we have to pay so much tax.’ [P28, female]
Preference	‘Most of the time like when I have like, since I go to school, I get my money, but like I don’t have enough to buy foods that I would want to eat every day, but I get to eat it once or twice a week what I love, what I would desire to eat.’ [P13, female]
	‘Well, I think it’s the fact that we don’t have enough for buy more healthy stuff.’ [P9, male]
Meal skipping	‘I spend all my week money before the week is over, I probably would go home and eat, or I would just like try to drink water to stay hydrated and I don’t eat during the day.’ [P13, female]
	‘Sometimes I eat like two times a day, but I make sure my kids eat three times a day. Yeah, so sometimes I eat three times a day. Sometimes two times a day…Like, especially Sunday. Sunday and Saturday. But during the week, I eat three times a day, because then I try to get for my kids, so we can have food for three times a day’ [P27, female]
	‘I usually make tortillas, which is basically the cheapest thing. Um, tortillas, bean, cheese, eggs. Um, the most cheapest thing that I can find, because most of the grocery items are much more expensive, especially chicken and canned foods and stuff like that. Yeah. On the weekend, when I’m at home, I would eat two times for the day. It’s only when I’m here at work I, you know, get to eat three times a day. But otherwise, I only eat two times a day, which would be lunch and supper. For lunch, I would cook rice, you know. It’s not all the time I can afford to get coconut milk to put in it, so sometimes it’s just rice and um I would make meatballs from oats.’ [P14, female]
Education	
Costs of tuition	‘Right now I have a lot of expense cuz I have four kids. I have three that are left school, but right now I owe the school for two of them, because one of my daughters she get help from some White people that come from States…And I don’t know, because it hard right now. No money. I can’t work, because if I work, there aren’t nobody to mind them. [P27, female]
	‘There’s many factors that causes a child not to get an education. Not saying it’s all, but the families that are big numbers, it’s harder for them. Now as a family of two, it’s easier; the parents could go out and find a job and put both students to school.’ [P25, female]
Attending a ‘good’ school	‘You know, if I came from [one school] and somebody else came from [a different school] and we went to apply for a job, best believe that other person will get that job, not me.’ [P14, female]
Employment	
No jobs	‘Sometimes people don’t have jobs, so it’s hard for them to make money to go buy their foods.’ [P10, female]
	‘They can’t find a job, there’s no jobs, so…I think that’s one of the biggest problems.’ [P11, female]
Jobs do not pay enough	‘In Belize it’s kind of rare for everyone to have a good paying job.’ [P12, female]
	‘Especially mom, sometimes she don’t have the food to give her kids, and that’s why that’s why she sometimes, she don’t make them eat even her eat no kind of food when they don’t have, because how she done in her fifties, so she cannot get a good job. She get a jobs, but they charge her, when she go to cleans to a place, they charge her just give her $20 or $25 and that is not a good money for they get food for the kid.’ [P15, female]
Cannot get a job because do not have a good enough education	‘Even here, so many people come and ask for a job, and they even say they’ll do it for a plate of food or, you know. There’s no job and especially if you don’t have an education, what are you going to do?’ [P11, female]
	‘I believe that the resources the government is getting, they’re putting it into building a street, infrastructure of streets and bridges. There is a few people that get contracts to do that. That specific job. Not everybody’s an engineer.’ [P22, female]
Transportation and distance	
Transportation difficulties	‘Well, there are villages here that are a few miles away, so if they don’t have access to come into town, they won’t be getting like the fresh produce….Well, they would need to get a bus or a taxi to like come into town or if they have a private vehicle or if they have a motorcycle, then they would come in with that, get what they need, and go back out. because gas is really expensive, so like to charter a vehicle might cost, you know, a great amount of money that they probably don’t have, so then I guess they would just do without.’ [P6, male]
	‘Mainly because it’s too far for them to get out, and some of them lack of transportation. And if they don’t have a transportation, so they don’t come out so much.’ [P16, female]

### Family composition as a barrier to food access

Though participants were not directly asked about their family composition, the theme of family makeup emerged, especially having a large family with many children, as a factor in accessing food. Some individuals had households with a significant number of children in the family, while others were led by a single parent or had only one working adult in the household. In nearly all instances, family makeup was tied to being able to afford food and provide for everyone in the household. This illustrative quote describes this phenomenon:

‘….when there’s too much members, too much siblings in a family, like for example in my family we are 14 kids and a single mother. And, uh, my mom had to, you know, work three jobs and it’s still not enough to feed the kids, so I would say a little bit of poverty in our community and the lack of not having both parents sometimes. It’s a part of the stress that it creates when you can’t bring enough food for the children or the children would not eat enough, because of the shortage of food that they have.’ [P26, Female]

In the case of single parenting or having a single provider in the family, participants described different circumstances that led to difficulty in accessing food. For example, some families experienced the death of a parent which influences the care of children:

‘One family, I would say is the family across from my house, my neighbor. They lost their mother last year, last October, and she left behind two small kids and their fathers, both of them drink, yeah. So, mostly we are the ones that get to feed them. It’s really unfortunate because there’s one that don’t have that support in the family.’ [P25, female]

Other families have experienced the incarceration of a parent and provider, influencing not only food purchasing but also food preference when they are left with little choice due to lack of money:

‘But right now, like I tell you, my husband go to jail, so I no buy food for them [the children]. But if there’s somebody that come and give them, yes….we eat it. We don’t pick, because we don’t have enough to pick and choose, so what they give us, we eat it….’ [P27, female]

Some households have experienced difficulties due to lack of assistance from one parent, which then compounded difficulties in accessing food:

‘You know, some of these teenage girls that are mothers, like they don’t have any assistance from the father of the child, you know, because of so many issues going on’ [P14, female]

### Income and financial status

A consistent theme around income and financial status appeared among interview participants influencing their ability to afford food. Some participants recognised that there are people in their community living in poverty which makes food access difficult in general:

‘Some people don’t have the money to buy the food, because, some people are really, really poor, poor. They can’t afford food.’ [P11, female]

Participants expanded on this and recognised that many people work for little wages, thus making it difficult to access foods:

‘Here in our country we work for little, a minimum wage, and when we go to buy, it’s too high for us. It’s why we can’t afford like everything.’ [P3, female]

Others cited increasing costs of living in Belize around services and goods as a contributing factor of poverty and food insecurity:

‘A lot of people live in the poor class of society in Cayo and in Belize on a whole. So is it more of the income. Housing has become a little more expensive that it was. Utilities have become a lot more expensive. Even gasoline and fuel has become very expensive in Belize. So, it is the income.’ [P1, male]

Many participants overwhelmingly went on to discuss taxes on foods:

‘The income of the family is not enough and the prices are high…Everything here has tax. And the tax is too high. And the income is low.’ [P11, female]

Additionally, there was some differentiation among participants in the ability to afford food that they preferred:

‘I don’t eat them [fruits and vegetables] daily, which is kind of sad but I do enjoy fruits a lot, but I cannot afford them, but they’re kind of like, they’re kind of pricey.’ [P5, female]

Some individuals also expressed how the inability to afford foods, or running out of money between paychecks, leads to meal skipping, cutting back on food between paychecks and ultimately food insecurity:

‘Okay, I do not provide for her [my daughter] as often as I would like. I would say two Fridays out of the month, we do not eat until nighttime after my husband gets his pay, because it isn’t enough with the rent and the bills and things….So, sometimes I have to cut back from me, so I could have for her to eat….’ [P25, female]

### Education

Education and access to education for children, in general, were also frequently mentioned themes, tied to future employment opportunities and higher income levels, which influenced ability to access and purchase foods:

‘the lack of um education, because if you’re not educated, now you can’t have a job. At first, you could get a job easily just by your experience, but now they call more the education level, so sometimes the kids don’t have education, but they have to work more hours to have more money to buy food for the family…’ [P23, female]

Participants noted that some families cannot afford the cost associated with school, including tuition and supply costs, resulting in dropout and limited employment opportunities:

‘There have been families in my community that I’ve seen that some of them, some of the kids, stop going to school, because the parents have no money to send them to school, and so they stop going to school, and they start selling goods on the street.’ [P14, female]

Participants also mentioned that some families cannot afford to feed their children either before or at school or pay for bus fare costs associate with travel to and from school:

‘Some people don’t have food to feed their kids to send them to school, so they don’t really want to send their kids on empty stomachs to school. And they, I would say, the bus fare to go to school. Sometimes, they don’t have that, too. [P21, female]

Furthermore, participants mentioned that there is a perception around reputations of schools throughout the country and how attendance at different schools can influence hiring opportunities:

‘And because like here, if you don’t have, even if you have a high school diploma, if it’s not a well-recognized school or a religious school, they look at it as just a piece of paper, like literally. Because then you have, uh for example [name of local school]. That’s a very good school, a religious school. And then you have um, I’d say [name of school in Belize City], I’m comparing to a school in Belize City. You know, if I came from [school in Belize City] and somebody else came from [local school] and we went to apply for a job, best believe that other person will get that job, not me.’ [P12, female]

Participants also noted that accessible and affordable education, supported by the government, is important to a society:

‘They’re [the government] not injecting capital into human resource, where people can get a better education at a more affordable cost, and they could become an asset to society in the future.’ [P22, female]

### Employment

Interview participants consistently discussed employment opportunities in Belize, citing various difficulties with gaining employment, and thus being unable to purchase foods. For example, in general terms, participants stated that there were not enough jobs available:

‘There is enough food, but there isn’t enough people who have the money to buy the food, because we have a lot of people that don’t have access to jobs.’ [P14, female]

Expanding on this, one participant described difficulties getting jobs due to migration into Belize:

‘There’s not enough jobs. So, I believe that we Belizeans, we live with what we have. If we have a job, we try to do our best, and we try to keep what we have, because it’s very hard to get a lot of jobs. We have lots of foreigners coming and they will do work for a lower price. And we Belizeans, that’s the reason that the Belizeans are not getting the jobs, because the foreigners come, no? And they get the job, because [they work for less].’ [P19, female]

Additionally, some participants described how jobs that are available do not pay enough to purchase needed food because prices are high:

‘Sometime you have to work hard and when you go to the store, the things [are] expensive. Sometimes the money not enough to buy foods. And that’s $12 for a whole chicken and need the rice. So, I see it hard like that.….it’s hard to get a job.’ [P27, female]

Tying multiple challenges together, individuals discussed how they were unable to get jobs because of the difficulty in getting an education, ultimately, resulting in being unable to afford foods:

‘….majority of the people in Belize here does not have jobs to support the family, so the foods are expensive…there’s a shortage [of jobs]…. Because we sometimes don’t have the…get the education. Like we don’t have. Belize you have, like most of the jobs you have here is more for people who went through college, and so maybe this is a poor country and majority of the children here don’t get that education and to go to finish college and get a job like that.’ [P17, female]

### Transportation and distance

Though all participants were residents of the Cayo district, there was a divergence between participants on issues around transportation and distance. For those living in the villages farther from the two major city and town centres (Belmopan and the twin towns of San Ignacio and Santa Elena), there were major issues in accessing foods as the majority of individuals rely on public transportation (buses or taxis) or bicycles:

‘lot of people in Cayo—myself included—live very far from the market and there’s only really one major market and that’s here near to us, where we are right now. People have to come all the way from the villages, and travel all the way on buses or even bicycles. Sometimes we walk all the way down here just to get food….I would say the pricing is good, but the consistency isn’t especially on the weekends where there probably are only two buses that run for a day, or three at max…For those persons who can have a ride, a lot of people hitchhike. A lot of people ride their bicycles and that’s a long, long journey…If you have a vehicle it’s easier, but most people don’t.’ [P1, male]

Though bus scheduling seems to have improved in recent years, individuals expand on the difficulties in accessing transportation to outlying villages around the major market:

Okay, right now they at this point in time, there are more buses than there were like, let me see, four years back maybe. To Santa Familia you get like a bus every hour. There’s a bus every hour. They go as far as Spanish Lookout…But, to the villages like rural villages, for example Cristo Rey and San Antonio, it would be more difficult for people, because there would be maybe one hour they only three schedules for the day. So, if you don’t really catch that bus at one, you have to catch the other one ‘til about three o’clock…’ [P2, female]

For the majority that do rely on public transportation, rough travel on unpaved roads is a challenge:

‘some of them live very, very far. Way in like bushes…we have to go all in those bushes, sometimes like little, like little roads to reach…Because I think maybe it’s too far or maybe it’s because the roads are bad, and one time even when we went back there, we just got stuck in the bus. The bus got stuck in the big hole with muds right there. We had to stay there ‘bout an hour trying to get it out. Then we were nowhere that had service for phone, nothing. We were like stranded ‘til we got it out.’ [P11, female]

Additionally, the expense of paying for transportation is another burden for individuals:

‘some of them don’t have access to transportation to get to these places. For someone that’s working, $2 isn’t much for a cab, $2 isn’t much. But for someone that’s not working, it’s very expensive, because in the city, the shuttle is a dollar, right, but they don’t have any dollar buses here, you know. So, it would be much harder.’ [P14, female]

Compounding difficulties with transportation in general, families with children experience financial and logistical challenges for their children travelling back and forth for school and lunch recess, as one individual described:

‘Yeah, some of them, the kids live very far out and maybe lunch time, they cannot go home for a lunch, because early in the morning, they have to get a bus bring them to school. Then at like 3:30 when they come out, they have to catch that bus to go back home, because they don’t have money to pay a taxi and they live far, like out in the, like some of them live in the bushes.’ [P3, female]

In contrast, for those living closer to the city or town centres where the main markets are located, described the ease of being able to walk to the market:

‘we are walking distance from the market, so we would go down to the market and buy whatever we’re going to use for the day, and that’s the way we eat. We eat the freshest of vegetables that we could get.’ [P18, female]

## Discussion

To our knowledge, this qualitative study is the first to provide contextual meaning around food security in the Cayo District, Belize, building from our previous quantitative findings and underscoring the importance of engaging in mixed-methods research^(27)^. Individuals highlight a complex and nuanced interplay of individual- and family-level barriers to food access, which ultimately drive and contribute to food insecurity. While these concepts are presented separately in the results, the conceptual map generated through group coding and thematic analysis shows the interplay between these barriers and their contributions to food insecurity.

In this present study, individuals cite difficulties affording food, having to stretch food among family members or missing meals until the next paycheck due to these various individual- and family-level barriers, all of which are hallmarks of food insecurity^(2,3)^. Participants indicate this is widespread and not localised to a small group of individuals, which is supported by income data in Belize. Poverty and its relationship to food access has been shown to be significant driver of food insecurity^(28)^. Exacerbating this are unemployment rates within in the Cayo District (13·6 %), which experiences the highest rates of all districts, compared to nationwide rates (9·4 %)^(29)^.

Though not specifically asked, nearly every individual placed family composition within the interaction between employment, income, and education and their broader relationship to food security. These qualitative findings complement previous findings^(19)^ and highlight the complexity of family composition and its contribution to food insecurity and malnourishment in a household^(30–33)^. Additionally, single-parent households, particularly female-led, experience additional burdens and greater vulnerability^(32,33)^. Family size in Belize is typically larger than family size in other Central American and Caribbean countries^(34)^ which may contribute to difficulties in accessing food. Specifically, family sizes in the Cayo District, especially in the rural areas, are larger than most districts, though these numbers are slowly decreasing^(35)^.

Compounding these matters, participants emphasise a troubling issue around access to education which is known to drive employment opportunities, earning power, and ultimately, food security^(36)^. Belize’s primary educational system (approximate ages 6–13) is compulsory and supported by public funds; however, there are significant fees associated with attendance. Both large and small families often struggle with paying fees and it is common for children to drop out before completion^(10,37)^. For children who can complete their primary education, families are then faced with significant burden of paying for secondary education, which is not free. Approximately 40 % of children aged 13–16 years in Belize do not attend secondary school, thus reducing employment opportunities and long-term earnings^(10)^. Specifically, the Cayo District has the highest proportion of children (30 %) not attending school^(35)^. Often, these children will not have necessary skills or meet employer educational requirements for hiring or advancement in many industries^(35)^.

Though study participants, who were primarily women, emphasised challenges around access to education and its relationship to food access, they did not describe gender differences in access to education. This is supportive of data showing that Belize experiences gender parity in primary schooling and recent favouring towards girls in secondary schooling^(35)^. Contrary to what so many other countries face in gender disparities around education, this offers some long-term hope in Belize that women will go on to tertiary schooling and have greater standing in higher paying jobs. Women with higher educational levels also tend to have smaller families which allows them to invest more in each child^(38,39)^. Additionally, women with higher nutritional knowledge have been linked to fostering a healthier home food environment^(39,40)^, since women and mothers are usually responsible for family meal preparation and food purchasing as food gatekeepers^(41–43)^. Certainly though, aligning with the United Nations Sustainable Development Goal 4, equitable and inclusive educational investment for all children and young adults will be beneficial in Belize to create a more qualified workforce and informed population. A focus on developing a more robust scholarship system or pay-gradient scale for tuition, while also investing in a national school-meal programme, would significantly ease burdens on multiple fronts for families.

This study confirms our earlier notion that transportation and distance to food sources and schooling are a significant contributor to food access and overall food insecurity in the Cayo District^(19)^. For those living in the town centre area, walking is readily accessible. However, for people living farther out, transportation challenges arise. Belize has several bus companies that operate in each district, but, as participants mention, routes are designated to main roads and run on limited schedules. Travel on rough roads makes a trip incredibly time consuming or nearly impossible, particularly during rainy season, when roads are muddy and bridges are washed out. Additionally, climate changes and recent climate events have been shown to have catastrophic impacts, resulting in areas being completely cut off from transportation and food deliveries and people fleeing and losing their homes^(44)^. The challenges around transportation and distance that our study participants cite are not unlike what other communities in the region face, and until there is considerable investment in public infrastructure, like road and bridge building and flood management, these challenges will persist^(45–48)^.

Data from this study were collected before the COVID-19 pandemic and our intent was to provide contextual meaning around food security. The pandemic has severely decimated the tourism industry in Belize that, for many, provides income and job opportunities. With mandatory lockdowns and travel restrictions, like what most countries have experienced, unemployment rates have soared^(49)^. School closures have severely impacted learning opportunities and access to school feeding programmes for thousands of children^(50)^. We believe the COVID-19 pandemic has only exacerbated issues described here and recovery will take years. Continuing this line of inquiry to understand the extent COVID-19 has impacted individuals and the food landscape in Belize will inform future economic and policy decisions.

### Limitations

There are several limitations to this study. First, interviews were conducted in the town’s centre, and therefore, we did not adequately gain perspectives from individuals living out in the rural villages beyond access to public transportation or within a reasonable walking or bicycling distance to the towns’ centre. We suspect these individuals experience even greater challenges around food security. Second, all of our participants were English speakers; therefore, future studies should be carried out with Spanish-speaking populations also, or individuals living in the Cayo District who are immigrants from other Central American countries. These individuals may be more at-risk for food insecurity.

## Conclusion

The findings of this study provide contextually rich descriptions of issues contributing to food insecurity among individuals in the Cayo District, Belize, namely individual- and family-level barriers to food access. Through group coding and thematic analysis, we have generated a simple conceptual map that describes barriers to food access and food security. Immediate attention to developing more robust educational scholarship and financial support systems would improve the situation on multiple fronts for individuals and families. Not only would it encourage attendance for children at school, but it would also impact and reduce other barriers that affect food security. Additionally, investment in public infrastructure would lessen transportation burdens to access food. However, future work is needed to explore Belize’s rich and ever-changing cultural, political and economic situation, both pre- and post-COVID-19 pandemic. Understanding these systematic challenges can help to situate the country in terms of population-level food security and provide avenues for change.
